# 

**Published:** 1877-01

**Authors:** 


### CONTENTS:

Article I.—Outline of Lecture. By Prof. Hodgkin, D. D. S. .... 385
Article II.—New York Odontological Society .... 389
Article III—How Best to Establish a Dental Practice. By Dr. E. G. Wheeler, D. D. S. .... 400
Article IV.—Virginia State Dental Association .... 408
Article V.—External Use of Salicylic Acid .... 410
Article VI.—Clinical Remarks on Cases of Acute Necrosis. By Mr. Savory, M. D. 414
Article VII.—The Treatment of Abscesses by Hyper-Dentition with Carbolized Water. By G. W. Callender, M. D. .... 417
Article VIII—The Old and the New. By Henry S. Chase, M. D. .... 419
Article IX—The Local Action of the so-called Astringents on the Blood-Vessels .. 421
Article X—Treatment of the Dental Pulp. By Lane Clark, D. D. S. .... 422

### EDITORIAL, ETC.

Vulcanite .... 424

Death from Ether .... 426

European Visitor .... 426

### MONTHLY SUMMARY.

Cure for Tetanus by Mechanical Measures .... 426

Is Alcohol a Food ? .... 427

Medical Women .... 428

Congenital Cleft Palate .... 429

Chloral Plaster in Neuralgia .... 429

Irritating Effects of Salicylic Acid .... 430

On the Local Action of Atropia .... 431

Deaths from Chloroform .... 431

Carbolic Acid as a Local Anæsthetic .... 432

Mastic Chewing .... 432

A Sensible Precaution .... 432

